# Visualizing joint force–velocity properties in musculoskeletal models

**DOI:** 10.1098/rsos.251066

**Published:** 2025-11-12

**Authors:** Christopher Richards, Tiina Murtola

**Affiliations:** ^1^ The Royal Veterinary College Department of Comparative Biomedical Sciences, London, England, UK

**Keywords:** musculoskeletal modelling, reaching, force–velocity properties, data visualization

## Abstract

Musculoskeletal modelling opens windows into how muscle properties interact with neural control to govern movement. Although musculoskeletal models produce vast computational data, they lack a visual language that compactly communicates how joint dynamics relate to time-varying muscle activation, force and length change. We developed a novel representation of joint-level force–velocity (joint-FV) properties, which shows how agonist and antagonist muscles contribute to the overall joint state and its trajectory throughout a movement. Using a model of human goal-directed reaching, we used joint-FV visualizations to discern the salient joint dynamic features across joints and between different reach targets. Regardless of target, we found that the shoulder, elbow and wrist joints traversed a near circular trajectory through joint-FV space when muscle forces were dominant, but trajectories were more complex when joint-interaction forces dominated (i.e. cross-joint forces due to Coriolis, Euler and centrifugal effects). Additionally, we found that co-contraction steepens the slope of the instantaneous joint-FV curve, causing damping, which helps stabilize against small perturbations. We therefore propose that our joint-FV visualization can be used to explain the intricate features seen in musculoskeletal simulation data to reveal how intrinsic muscle properties govern the behaviour of dynamical systems.

## 1. Introduction

Musculoskeletal modelling is a powerful tool for understanding how physiological and anatomical properties of muscles not only enable versatile behaviour [1,2], but also limit locomotor performance [3]. Profound technical advances, such as open-source physics engines [4] and optimization techniques [5,6], have enabled broad exploration of musculoskeletal biomechanics both in humans and in other animals [7]. However, the generation of fundamental knowledge from musculoskeletal modelling approaches is not necessarily straightforward given the richly detailed data they produce. Thus, despite the wide availability of modelling tools [8,9], there remain gaps in our detailed understanding of how muscle dynamics interact with the intricate physics of biomechanical systems.

A primary reason why simulated data from muscle models can be challenging to analyse and visualize is that they churn out multiple streams of time-series data (e.g. force, length, velocity, activation and force–velocity gain). In particular, the question, *What is the functional role of muscle X?* is often difficult to address thoroughly; although computational methods for discerning patterns do exist, visualization of those patterns is often cumbersome. This is because of the great demands of interpreting large numbers of time-series plots and understanding interactions among them. Nevertheless, visualization has long been an important tool for communicating key structural properties of time-series data [10]. Moreover, novel visualizations can further drive the generation of knowledge, particularly with dynamic biomechanical systems (e.g. visualization of skeletal movements using XROMM [11]).

Fortunately, a significant advance came from plotting muscle force against displacement to create a ‘work loop’ figure [12,13], which collapses muscle dynamics into a trajectory whose shape and direction visually communicates function; i.e. whether the muscle acts like a motor, brake, strut or spring [14–16]. To probe further, one can then address the question, *How does the muscle achieve its function?* This is done by observing where the muscle operates on its force–velocity (FV) curve (see [15]). For example, during early stance in human running, ankle extensors achieve ‘strut-like’ function due to their operating range on the FV curve during high activation [17]. Furthermore, FV effects (in the form of gain functions) can be plotted as time series to demonstrate the relative impact of activation versus length versus velocity effects on the temporal features of force production during dynamic activity [18,19]. Unfortunately, achieving this level of functional and mechanistic detail requires several plots for each muscle. Hence, these types of zoomed-in analyses of muscle functional mechanics are often restricted to ‘simple’ models (i.e. one or a few muscles; e.g. [20–23]), whereas a similarly detailed mechanistic analysis for complex musculoskeletal models (e.g. [2,24–27]) is not always practical, given the number of plots required.

In addition to the data presentation problem, musculoskeletal models have many interacting levels of dynamic behaviour that are difficult to digest. At one level, musculoskeletal models are digital marionettes made of linked segments that can behave counterintuitively and chaotically due to cross-joint interactions (e.g. [28]). These multibody systems require extensive nonlinear equations of motion (e.g. [29]) which make intuitive grasp of the physical behaviour challenging. At another level, muscle properties such as force-length-velocity and activation dynamics introduce additional layers of nonlinear behaviour (e.g. [30]), which might grow even more difficult to comprehend during co-contraction. Hence, a holistic and thorough understanding of musculoskeletal dynamics is profoundly challenging to reach and communicate in a single study.

To help overcome the impracticalities of analysing arbitrarily complex musculoskeletal models, we aimed to consolidate muscle-mechanical data into a compact visual format. We introduce a new conceptual framework that visually encapsulates multiple streams of data onto a single plot. We propose a visualization of ‘joint-FV space’, which consolidates muscle data from agonist and antagonist muscles to create a portrait of joint mechanical function. We then use joint-FV plots to unravel how joint-level behaviour is explained by mechanical interactions among antagonistic muscle pairs.

To demonstrate our new framework for visualizing muscle-mechanical data, we explore the detailed muscle dynamics of a human reaching model [31], which encapsulates all the challenges discussed above. We pose three questions: (i) Do the three arm joints function similarly to each other during reaching? (ii) Does each joint function similarly across different reaching motions? (iii) How does co-contraction influence joint-level FV properties? We found that although generic answers to these questions could be discovered from traditional time-series plots, additional details would have been missed without a visualization of FV properties at the joint level. Thus, we propose that our novel framework takes one step towards tackling the challenge of understanding muscle dynamics within musculoskeletal models.

## 2. Theory and methods

### 2.1. Constructing the joint-level force–velocity space

**Figure 1.**
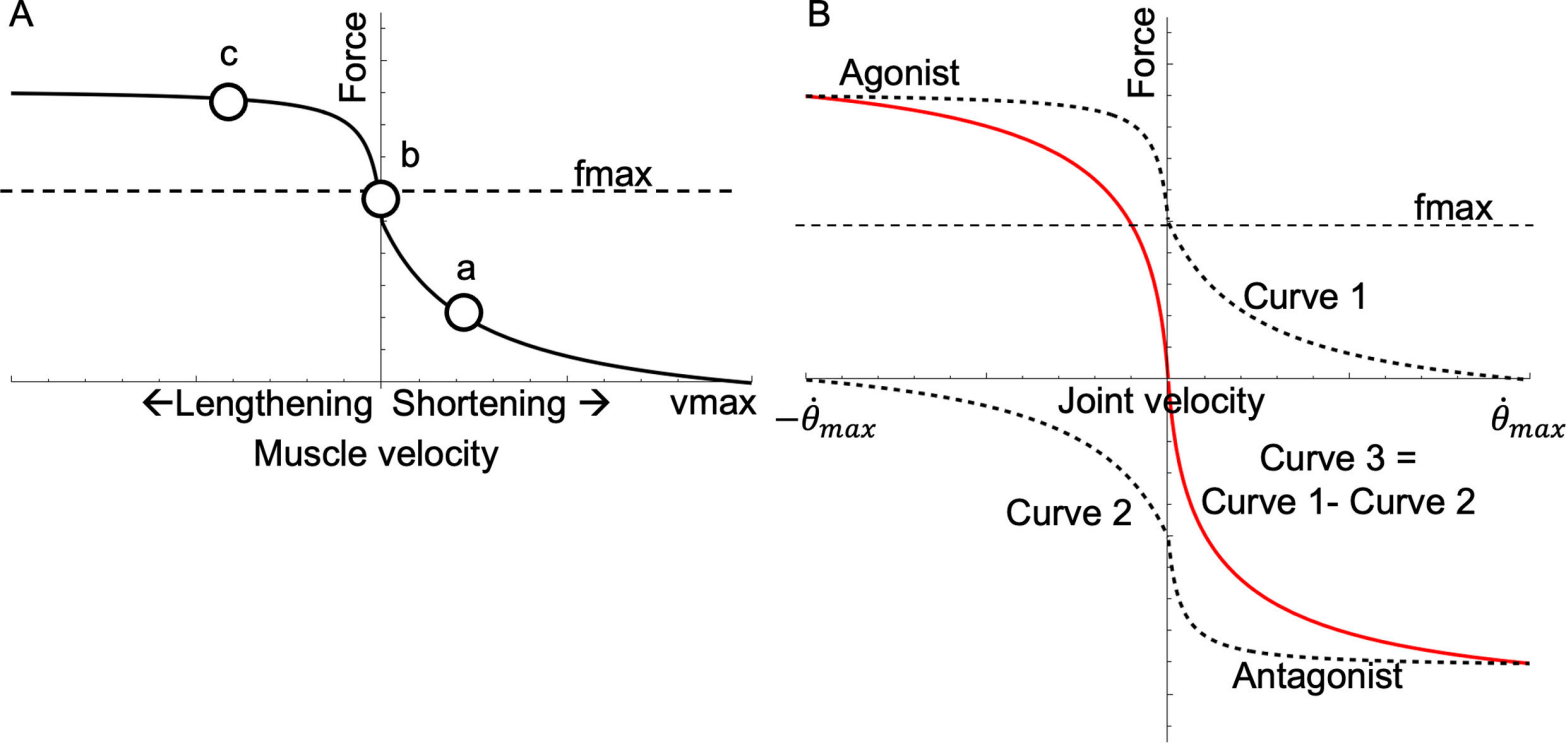
The FV relationship. Muscle force falls hyperbolically with shortening velocity but increases steeply with lengthening (A). Much of dynamic muscle function can be understood by a muscle’s operating point on its FV curve. The diagram shows three hypothetical operating points on the curve (white circles). Point a represents a muscle shortening at approximately 1/3 vmax, which is optimal for mechanical power (e.g. a muscle at optimal cycling frequency such as in a swimming fish, e.g. [20,32] or a bird at takeoff, e.g. [33]) versus point b where the muscle operates isometrically (e.g. a leg muscle in a running turkey remains isometric while work is performed by the tendon [34]; versus point c where the muscle resists lengthening to absorb energy (e.g. a stabilizing muscle of a running cockroach; [14,15]). Joint–fv relationship (B). The FV curves of the agonist (curve 1, upper, dashed) and antagonist (curve 2, lower, dashed) are identical in joint velocity space, but reflected about the *x-* and *y*-axes. The agonist curve intercepts the *x*-axis at +${\dot{\theta }}_{max}$, whereas the antagonist intercepts at −${\dot{\theta }}_{max}$. Subtracting curves 1 and 2 gives the joint–fv curve 3 (solid red). Note that the joint–fv curve (curve 3) has local regions of steeper slope than either of its component curves, especially near the origin.

In a traditional Hill-type muscle model, the familiar FV relationship is one of the key features that governs the dynamic behaviour of a muscle-mechanical system. Although the force–length (FL) relationship might play an important role in slow movements, for the current study, we assume FV effects to be dominant (see Discussion) because of the substantial velocities and time-varying loads during reaching [31]. In Hill-type models, the FV relationship is drawn as a single curve representing maximum activation such that the normalized muscle force reaches 1 (i.e. maximum force) when velocity is zero (figure 1A). Equivalently, the FV relationship can also be understood as a space between two curves where the FV curve defines the ceiling (maximum activation), and the *x*-axis is the floor. The muscle operates on or between these boundaries. Specifically, when activation is submaximal, the Hill-model FV curve is scaled downwards. Thus, FV space is made from an infinite number of lowercase ‘fv’ curves representing different levels of activation. Consequently, a muscle’s functional state may move through FV space in two ways: (i) by travelling along its current fv curve (constant activation) due to changing load velocity or (ii) by moving across fv curves (changing activation). During functional behaviours, a muscle generally moves from one region of the space to another. For example, a muscle which shifts between acceleration (e.g. jumping) and stabilization (e.g. landing) moves from a region of high power (i.e. approx. 1/3 maximum contraction speed) to near isometric. How quickly a muscle’s state can change depends on load as well as the shape of the FV curve and the rate of activation change (as measured by the rising and falling slopes of isometric twitch or tetanic contractions).

**Figure 2.**
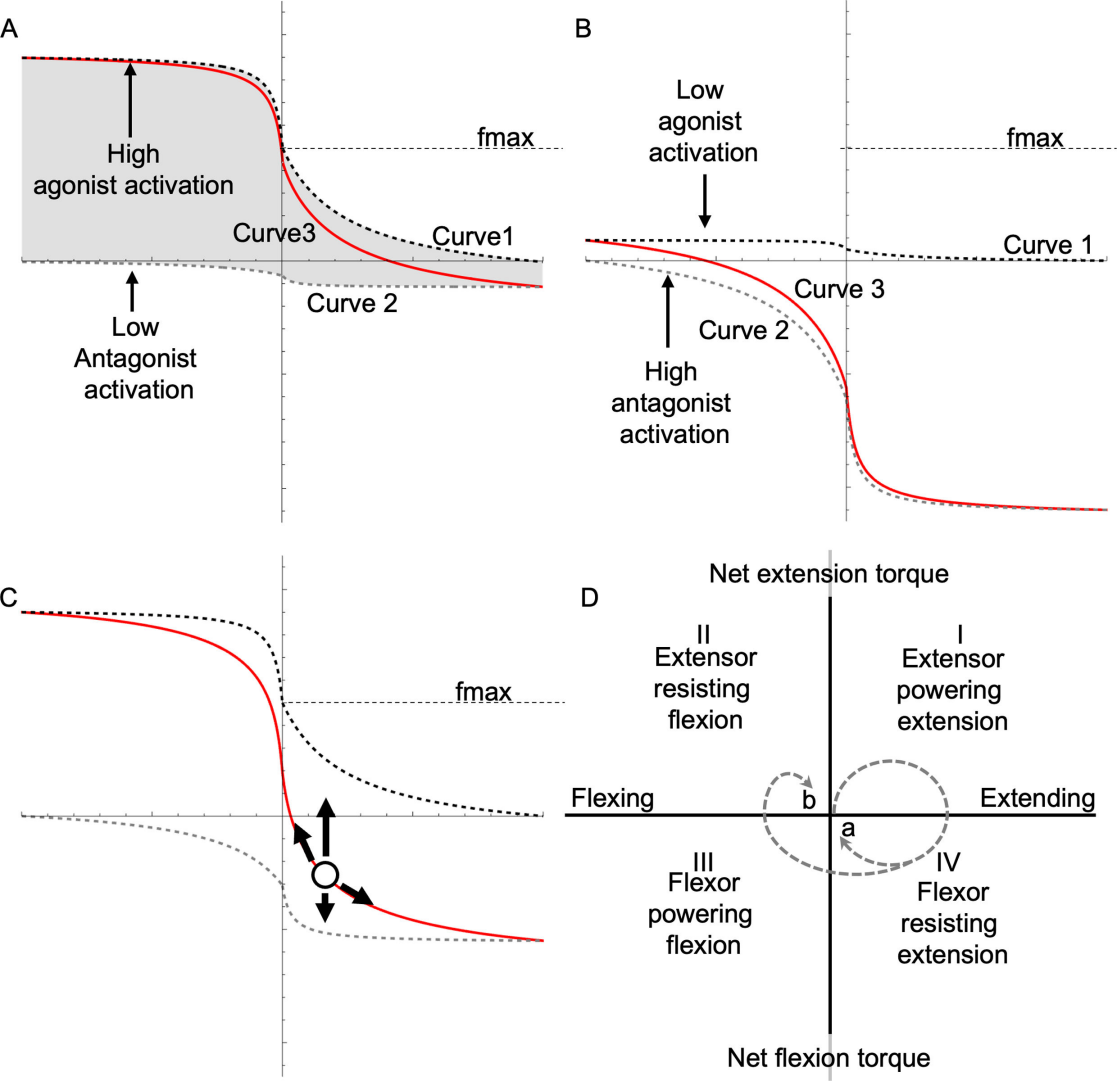
The joint force–velocity (joint–fv) relationship. (A) Curves 1 and 2 represent the upper and lower bounds of the joint–fv space, depending on the respective levels of agonist and antagonist activation. For any agonist/antagonist activation level, the area between curves 1 and 2 (shaded region) represents the ‘joint–fv space’. At a moment in time, the relative agonist/antagonist activation levels determine the joint’s instantaneous fv curve (red; curve 3). In this example, agonist activation is near maximum and agonist activation is near 0; curves 1 and 3 nearly overlap one another (they will entirely overlap if antagonist activation is 0). (B) In this reciprocal example, agonist activation is very low (compared with the antagonist), hence curves 2 and 3 nearly overlap. (C) This example shows maximum agonist activation, but 50% antagonist activation. The joint operating point (white circle) always falls on its instantaneous joint–fv curve. This point may move either up or down, due to changes in either muscle activation, or the fact that the point may move along the curve due to changes in mechanical loading. (D) The four quadrants of joint FV space indicate the mechanical function of the joint (e.g. with respect to flexion or extension action). In an ideal reach where the arm produces a monotonic movement precisely to the target, a joint would start from rest at the origin then move along a circular path (dashed path a). More typically, a joint trajectory has an additional homing-in phase (path b). In any case, the movement of the joint through quadrants reflects the time-varying mechanical function of the joint (see text). Note this example shows an extensor as the agonist (e.g. elbow, wrist); however, that choice is arbitrary; for the shoulder, the flexor would be the agonist and the flexion/extension labels would swap (see text).

While single-muscle contraction characteristics are governed by its FV relationship, musculoskeletal behaviour depends on how the FV relationships of antagonistic muscle groups interact at the joint level. We therefore introduce the concept of a ‘joint–FV space’, which describes all combinations of joint net force and joint velocity that are possible given the FV relationships of the muscle groups. In the general case with multiple mono- and biarticular muscles crossing a single joint, the shape of the joint–FV space is complicated. However, in the present work, we focus on the simplest case of a symmetric monoarticular joint with a single pair of identical antagonistic muscles (more complex configurations are possible, but outside of the current scope; see §4). In this simple case, the joint–FV space can be constructed by considering how the velocities of the two muscles are linked through the joint angular velocity. Consequently, the boundaries of possible joint–FV states are defined by the FV curves of the two muscles, where the antagonist curve has been reflected about both the *x*- and *y*-axis (because if one muscle shortens, the other must lengthen at the same speed, and the forces/torques of the two muscles act in opposite joint directions; figure 1B). The position of a muscle pair in this joint–FV space depends on the activation levels of the two muscles. Specifically, these activation levels define the current joint–fv curve (analogous to the single-muscle fv curve) and a local joint–fv space that spans the family of joint–fv curves between the two single-muscle fv curves (i.e. shaded region in figure 2A). The joint–fv curve represents all joint states accessible without changing activation levels, while the joint–fv space gives a visual representation of how each muscle contributes to the joint–fv curve. The morphing of joint–fv space creates a rich variety of possible trajectories through the joint–FV space as activation levels dynamically change during complex behaviours. Notably, when co-contraction occurs, the joint–fv curve differs in shape from the single-muscle fv curve, particularly near the *y*-axis, where co-contraction causes a steeper slope. Potentially, joint–fv plots can be useful for visually indicating the mechanical state of the joint. Specifically, during co-contraction, the local slope of the instantaneous joint–fv curve can theoretically increase damping (i.e. resistance to changes in velocity), which could impact joint stability.

The FV calculations are as follows. The agonist muscle FV curve is converted to joint angular velocity from the FV function used previously [23,31].


(2.1) $${\text{FV}}_{\text{agonist}}=\left\{ \begin{array}{ll} \;\;\;\frac{1-\tilde{\overset{˙}{\theta }}}{1+d_{1}\tilde{\overset{˙}{\theta }}}, & \text{if}\;\tilde{\overset{˙}{\theta }}>0\;\left(\text{shortening}\right)\\ d_{2}-\frac{\left(d_{2}-1\right)\left(1+\tilde{\overset{˙}{\theta }}\right)}{1-d_{3}\tilde{\overset{˙}{\theta }}}, & \text{otherwise}\;\left(\text{lengthening}\right) \end{array}\right.$$

where *d_1_*, *d_2_*, *d_3_* are shape constants (*d_1_* = 4, *d_2_* = 1.8, *d_3_* = 30.24 in the current study; [31]) and $\tilde{\dot{\theta }}$ is the normalized joint rotational velocity, which is positive for shortening


(2.2) $$\tilde{\overset{˙}{\theta }}=\frac{\overset{˙}{\theta }}{{\overset{˙}{\theta }}_{\text{max}}}$$

(2.3) $${\overset{˙}{\theta }}_{\text{max}}=\left(\frac{1}{r}\right)v_{\text{max}}$$

where *v*_max_ is the maximum muscle shortening velocity (in m/s) and ${\overset{˙}{\theta }}_{\text{max}}$ is the maximum joint velocity (in rad/s), given the moment arm, *r*.

The antagonist FV curve is defined as above, except with all velocity terms multiplied by −1 in equation (2.1). In reality, moment arms may be time varying and differ between agonist and antagonist. For simplicity, and to match our simulations, we assume agonist and antagonist moment arms are equal and constant for the current study; however, the framework can be expanded in the future to allow for these complexities (see §4).

The joint–fv curve (at one instant in time) is as follows.


(2.4) $$fv_{joint}=a_{\text{agonist}}\cdot FV_{\text{agonist}}-b\cdot a_{\text{antagonist}}\cdot FV_{\text{antagonist}}$$

where *a*_agonist_ and *a*_antagonist_ are the activation levels of the agonist (i.e. the dominant muscle) and antagonist, and *b* is a weighting factor, calculated as the ratio between maximum isometric antagonist and agonist torques to account for different muscle strengths and moment arms. The joint–fv space is defined as all the joint–fv curves equation (2.4) that correspond to agonist activations between 0 and current *a*_agonist_ and antagonist activations between 0 and current *a*_antagonist_. Note that the independent activation levels *a*_agonist_ and *a*_antagonist_ can equivalently be expressed using co-activation, which describes the level of activation simultaneously present in both muscles, and net activation, which describes how much excess activation the agonist has relative to the antagonist: *a_net_ = a*_agonist_ *– b·a*_antagonist_.

To generalize equation (2.4) to a variety of tasks, we redefine agonist/antagonist in a way that is independent of specific anatomical movement directions, such as flexion/extension. As the mechanical function of a joint is indifferent to anatomical orientation, we can freely choose which muscles to label as agonists based on the mechanical task. For some tasks, this selection can easily be made to align with anatomical convention, such as during a jump requiring leg extension, where all extensors would be called agonists and flexors would be antagonists. However, reaching is less straightforward because some joints may move in different directions (e.g. elbow extends whilst shoulder flexes) and the direction of joint movement may change during a reach (e.g. during homing-in), causing muscle roles to alter between powering movement, breaking, and stabilizing. Furthermore, this pattern may change depending on target location. To address this problem, we define an agonist as the dominant muscle that produces the power to accelerate a load during the first 0.05 s of the reach. Furthermore, we define the FV coordinate system so that the agonist FV curve is plotted with its shortening portion in the first quadrant of Cartesian coordinates, whereas the shortening portion of the antagonist appears in the third quadrant (figure 2D).

### 2.2. Visualizing the joint-force–velocity curve and space

Because of the asymmetry of the FV function, the shape of joint–fv space depends on the relative activations of agonist versus antagonist. At one extreme, the agonist contracts while the antagonist is inactive, causing the joint to operate on the ceiling of the space (on curve 1, figure 1B). Towards the other extreme, the agonist activation level is low, whereas the antagonist activation is high, such that the joint operates on the floor of joint–fv space (on curve 2, figure 1B). For any other activation values, the joint operates in the area between the ceiling and floor along an intermediate joint–fv curve (curve 3, figure 1B; analogous to the fv curves in the single-muscle example above). As the relative activation levels change, the joint–fv space morphs as the space between curves shrinks or expands to represent how each muscle contributes to the joint–fv curve (compare figure 2A vs. 2B). For example, low antagonist activation gives rise to a joint–fv space that is mainly above the *x*-axis, while a joint–fv space with similar areas above and below the *x-*axis indicates a high level of co-activation. Similar to how a single muscle always operates at a point along an fv curve, the joint always operates at a point in FV space along a joint–fv curve (figure 2C).

### 2.3. Functional interpretation of trajectories through joint-level force–velocity space

The current representation of joint–fv space is analogous to the ‘work loop’ concept of muscle contraction [12,13]. A work loop plot visually communicates muscle function because the work loop shape and direction indicate whether a muscle is producing mechanical work or behaving like a strut, brake or spring (see [15]). Analogously, the current representation of the joint-fv space can visually communicate joint function through the lens of physiological limitations of activation and FV properties. Specifically, the joint–FV coordinate system can be subdivided into four Cartesian quadrants (figure 2D). For illustrative purposes, we describe the quadrants in terms of flexion and extension, as follows. If the agonist is an extensor and the antagonist is a flexor, quadrant I represents extension powered by extensors. Quadrant IV represents extension but is resisted by co-contracting flexors. Quadrant III represents flexion powered by flexors. Finally, quadrant II represents flexion but is resisted by co-contracting extensors. Importantly, all muscles can be active in all quadrants, but extensors dominate in quadrants I and II, whereas flexors dominate in III and IV. When the joint operates along the *y*-axis, both muscles are isometric whilst the joint is stationary. The *x*-axis represents the balance of both muscles, meaning no net torque on the joint. For example, the elbow of a jumping frog would likely operate mainly in quadrant I during launch, but in quadrant II during landing. Following our current definition of agonist and antagonist muscles, the coordinate system (figure 2D) can be oriented to match the behaviour of interest. For example, a predominantly flexion-powered movement such as a ‘biceps curl’ exercise would have flexors occupy quadrants I and II (versus extensors in III and IV).

The dynamics of joint function can be studied by observing the trajectory of movement through joint–FV space. Similar to the single muscle example, a joint’s operating point can move anywhere in the available space either by moving up and down due to relative changes in agonist/antagonist activation or left and right along the current joint–fv curve due to changes in loading. Specifically, a joint’s operating point in FV space can move straight up as rapidly as the agonist activates (and the antagonist deactivates). However, a joint’s motion along the joint–fv curve is only limited by how rapidly the load can accelerate. For example, a joint working isometrically against a latched load will be on the positive *y*-axis; once the latch releases, the joint operating point will travel rapidly to the right along the joint–fv curve.

To summarize the above, several features can be immediately read from joint–fv plots. Similar to a work loop plot, one can see whether individual muscles are functioning as motors, brakes or struts by observing their operating points on their respective fv curves. More significantly, one can also read whether the entire joint functions as a motor (or brake or strut) and follow how this function shifts in time. Furthermore, one can read how this joint function relates to anatomical function by observing where the joint operates among the four quadrants.

### 2.4. Reaching simulations

**Figure 3.**
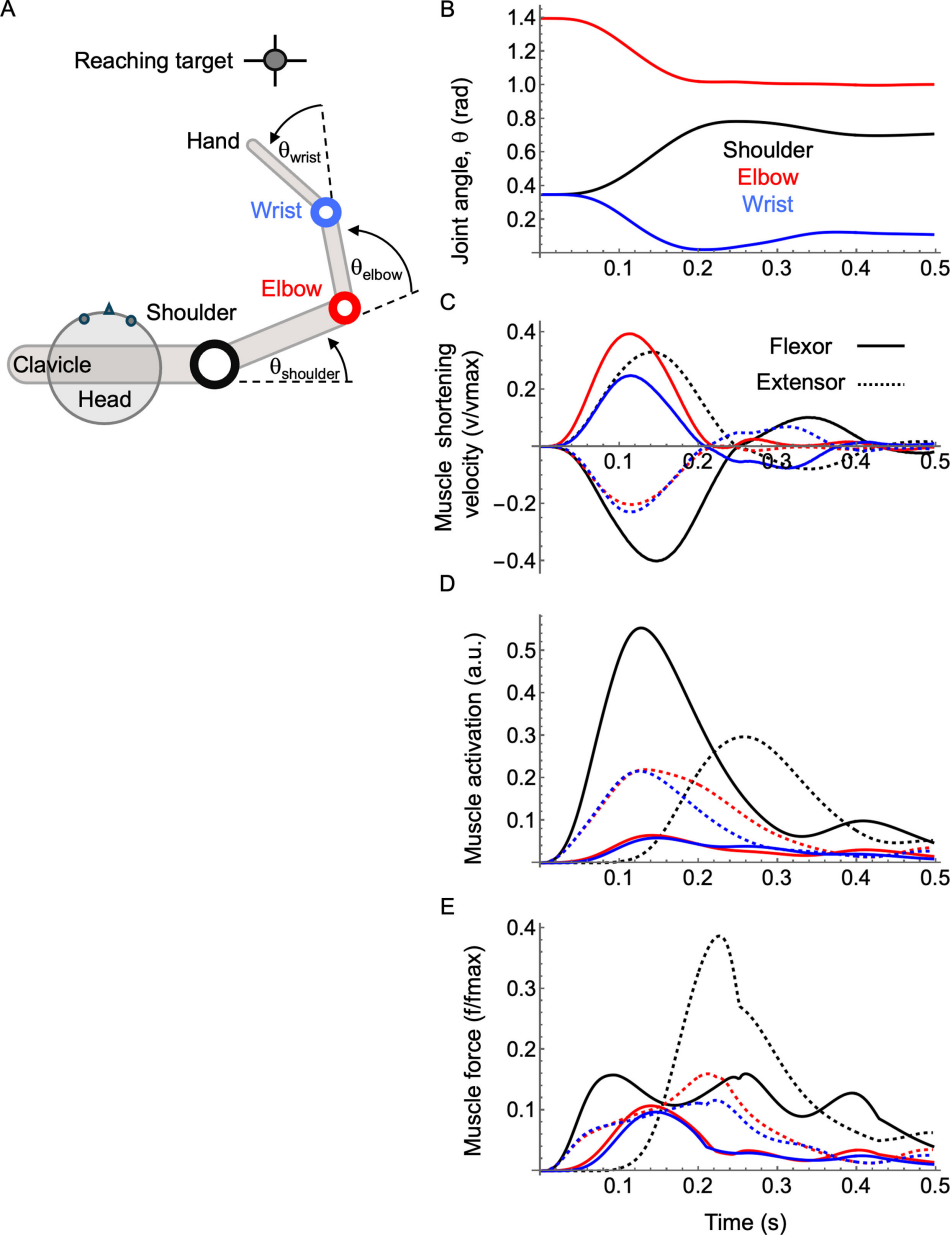
Musculoskeletal simulation of human goal-directed reaching. (A) A schematic of the horizontal reaching model (see [31]) consists of an antagonistic pair of muscles about each of its three joints (shoulder, elbow and wrist). Time-series data from a simulated reach showing joint angle (B), shortening velocity (C), activation (D) and normalized force (E) for muscles at the shoulder (black), elbow (red) and wrist (blue). Flexors are shown with solid lines, whereas extensors are dashed.

To demonstrate the basic features of the joint–FV concept, we first used a simple single-joint example. We then utilized a previously published musculoskeletal model of human goal-directed reaching [31] to illustrate the usage of the joint–FV space in a more complex musculoskeletal configuration. Briefly, the reaching model is based on simplified human anatomy and inertial properties, and it is restricted to the horizontal plane (figure 3A). The movements of the hinge joints at the shoulder, elbow and wrist are each driven by a pair of monoarticular Hill-type muscles. The arm is guided towards the target by muscle excitation signals computed by a PD controller following a pre-defined trajectory. All muscle properties are identical to our previous study; however, the parameters of the PD controller were retuned for rapid reaching (i.e. to reach the target within approx. 0.5 s).

To represent reaching over a range of conditions, we selected four conditions to compare: (i) forward target; (ii) sideways (ipsilateral) reach; (iii) cross-body (contralateral) reach; (iv) forward target, identical to (a), but with an external perturbation. The perturbations were modelled by injecting a 100N point force (approx. 1/20th the force of a punch; [35]) to the hand segment delivered at 90° anti-clockwise to the instantaneous hand velocity vector, approx. 150 ms after the start of the reach. We were careful to use a perturbation small enough to ensure the perturbed muscle speeds did not exceed *v*_max_ to avoid violating the assumptions of the Hill-type muscle model. Our code allows for arbitrary control of perturbation magnitude, direction and timing; however, a full sweep of perturbation parameters is beyond the current scope (see §4).

## 3. Results

### 3.1. A single-joint muscle-driven example

**Figure 4.**
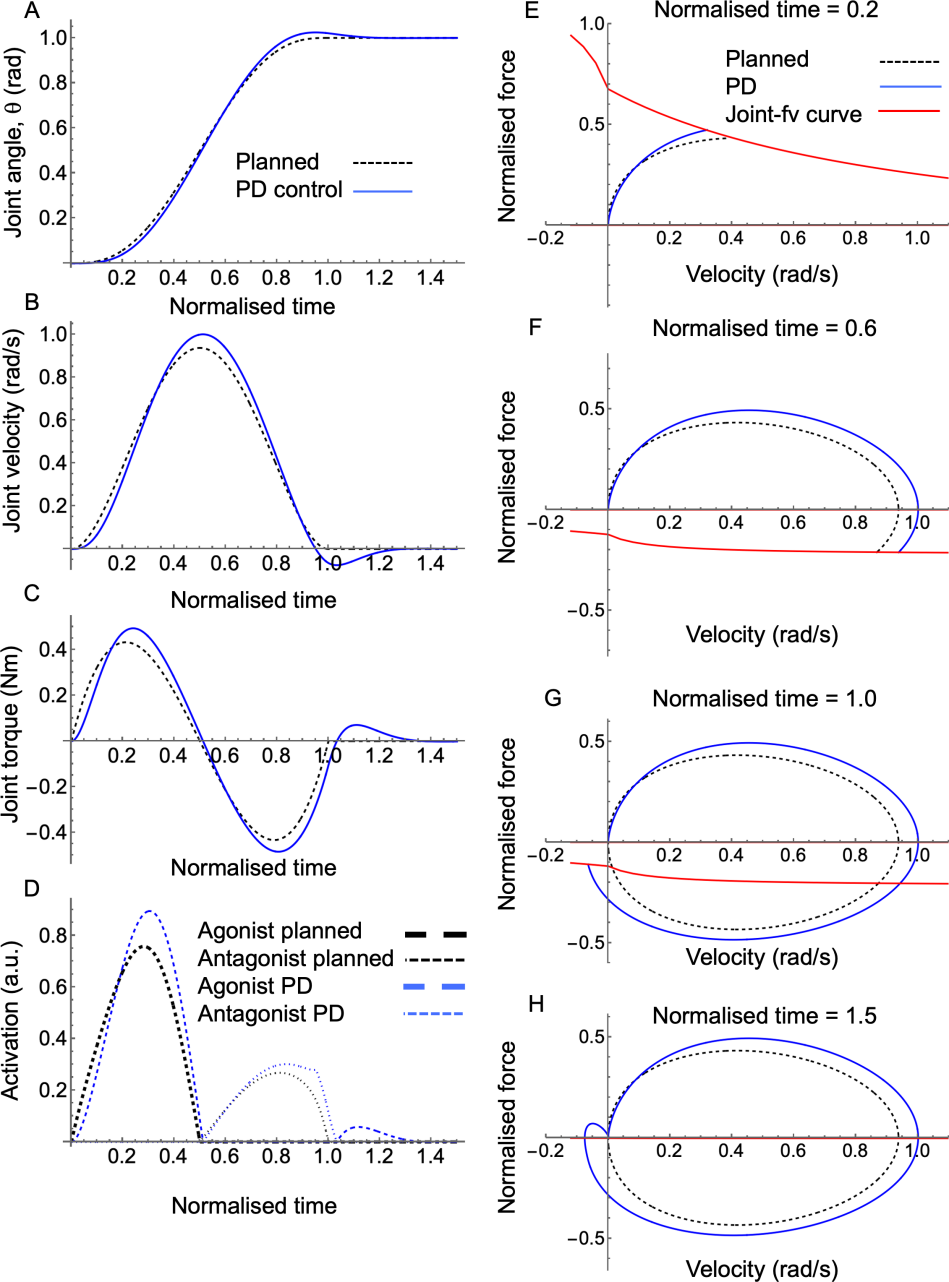
A single joint, two-muscle joint–FV toy example. Time traces of joint angle (A), joint velocity (B), joint torque (C) and activation levels for the two muscles (D), as well as joint–fv trajectories at four time points (E–H). All panels show the planned analytical solution (dashed black) and a solution obtained by tracking the plan with a PD controller. FV space panels also show the instantaneous joint–fv curves for the PD solution (red).

To illustrate the construction and inherent properties of the joint–FV concept, figure 4 shows a toy model of a single-joint movement with an idealized bell-shaped velocity profile driven by a pair of identical antagonistic muscles. If the planned movement is executed perfectly (i.e. following the bell-shaped profile), the joint velocity and torque time traces (figure 4B,C) form an egg-shaped joint–fv trajectory in quadrants I and IV. However, an imperfect execution (here obtained by tracking the planned trajectory with a proportional-derivative controller) causes the trajectory to deviate from the plan, resulting in tri-phasic activation of the muscles (figure 4D) and a corrective loop in the joint–fv trajectory (figure 4H). As there is no co-contraction in this simple example, the joint–fv curve is solely the fv curve of the activated muscle, and its relative flatness indicates that local changes in muscle force and joint torque production are largely driven by changes in muscle activation. This simple example illustrates the significance of the shape of a joint–fv trajectory; a round trajectory (egg-shaped in this case) indicates more ideal tracking, whereas deviations from this trajectory indicate imperfect tracking or external forces (e.g. perturbations). As will become clear below, reaching behaviour is more complex due to simultaneous movements among segments; these generate multibody forces causing joint–fv trajectories to deviate from the ideal case.

### 3.2. The muscular dynamics of forward reaching

The forward reach is a simple countermovement between the upper arm and lower arm where the shoulder flexes while the elbow and wrist extend smoothly towards the target (figure 3B; electronic supplementary material, Video S1). The movement has two phases: (i) the primary phase, where the arm gets close to the target, followed by (ii) a final correcting phase, where the hand homes in on the target. This is most clearly visible in the ‘two-hump’ pattern of muscle shortening velocities (figure 3C). The muscle activation patterns (figure 3D), however, are more complex and marked by strong co-activation and therefore co-contraction of flexors/extensors at all joints (figure 3E). The intricate pattern of muscle forces stems from the inherent complexity of multibody systems, where segment rotational velocities and accelerations are mutually dependent through nonlinear relationships. That is, some features of any given force profile are muscle contractions aimed at accelerating its respective segment, whereas other features work to counteract interaction forces between segments. Regardless of the complexity of force patterns, co-contraction is a salient feature across a broad range of reaching movements due to the intrinsic delays of realistic twitch and tetanic activation profiles, as represented by the third-order dynamics used in our model [31].

### 3.3. Joint-level force–velocity dynamics of the shoulder, elbow and wrist during forward reaching

**Figure 5.**
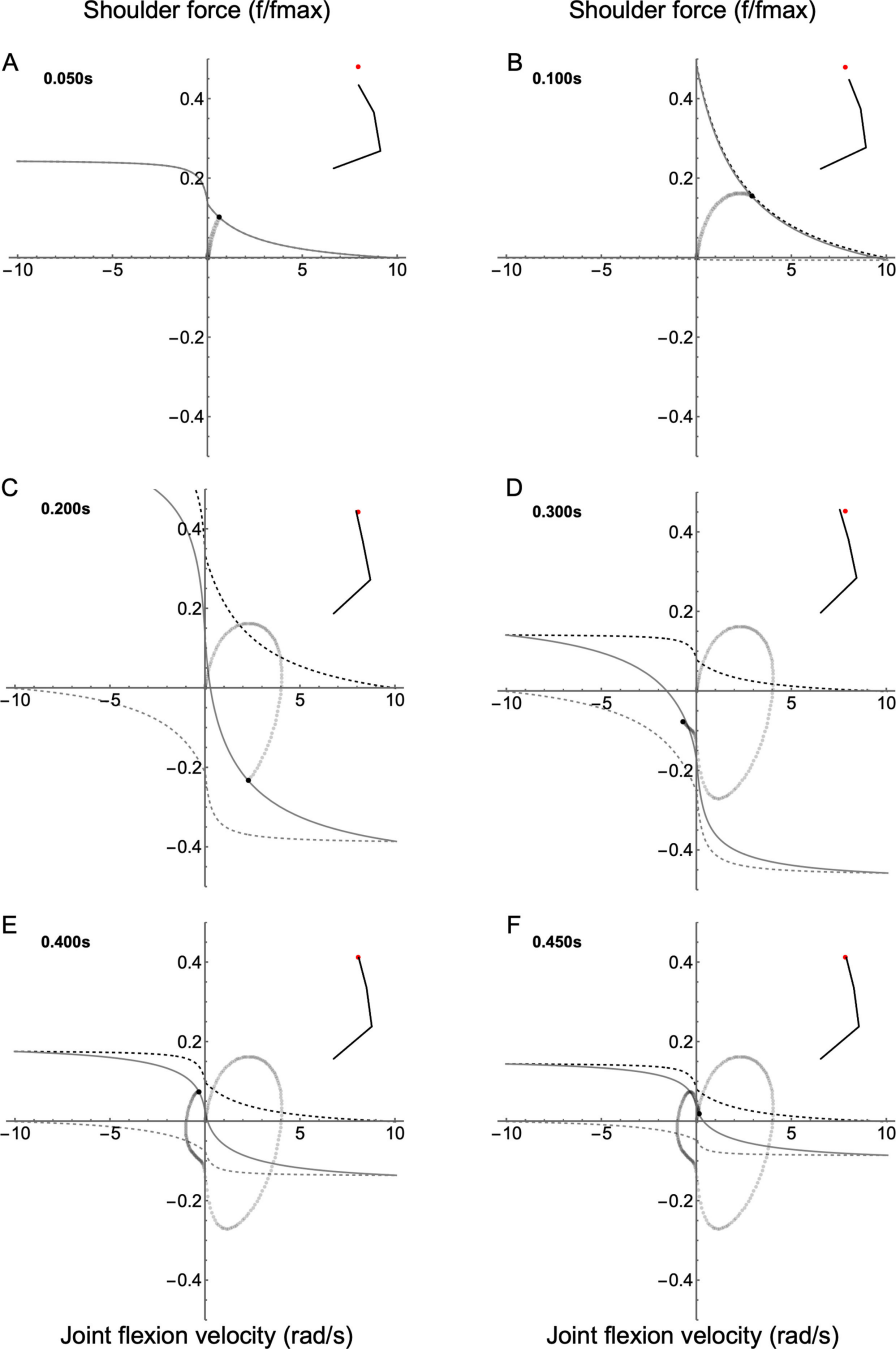
Joint-FV space animation for the shoulder joint. FV curves are shown for six time frames throughout the reach (A–F). For the shoulder, the flexor is defined as the agonist; therefore, positive force causes flexion. The inset shows the position of the right arm relative to the target (red dot). The joint state trajectory is shown as a trail of grey dots, with the current state indicated by the black dot. The solid black line is the current joint–fv curve, and upper and lower limits of the joint–fv space are indicated by the dashed black and grey lines, respectively.

Because the simulations start at rest, all joint trajectories begin at the origin of the joint–FV coordinate system. For the shoulder, the dominant action is flexion; therefore, the flexor muscle (agonist) drives the early part of the reach (figure 5A; electronic supplementary material, Video S3). As agonist activation rises, the joint–fv space expands and the joint travels along a parabolic trajectory until net activation reaches a peak (figure 5B) and then subsequently falls due to rising antagonist (extensor) activation. To slow the arm and minimize overshooting the target, the extensor then resists the flexion (figure 5C). In the final stages, the activation of both muscles falls, shrinking the size of the joint–fv space as the joint moves from flexion to extension to correct overshoot and home in on the target (figure 5D,E,F). Overall, the trajectory is a clockwise path through FV space (similar to ‘path a’ in figure 2D).

**Figure 6.**
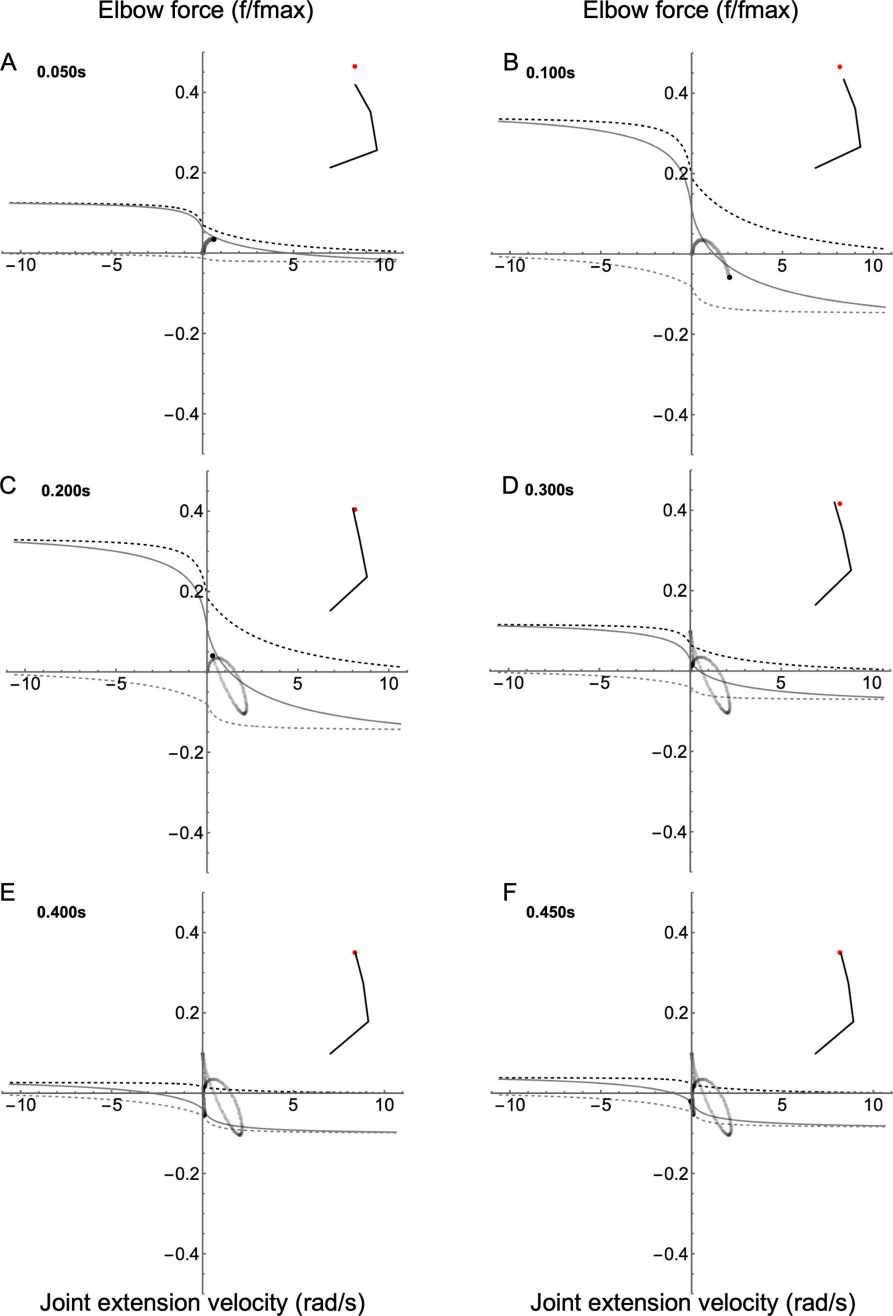
Joint–FV space animation for the elbow joint. FV curves are shown for six time frames throughout the reach (A–F). For the elbow, the extensor is defined as the agonist; therefore, positive force causes extension. The inset shows the position of the right arm relative to the target (red dot). The joint state trajectory is shown as a trail of grey dots, with the current state indicated by the black dot. The solid black line is the current joint–fv curve, and upper and lower limits of the joint-fv space are indicated by the dashed black and grey lines, respectively.

Similar to the shoulder, the elbow joint–fv space begins small (figure 6A; electronic supplementary material, Video S3), then expands to a peak (figure 6B,C), then falls throughout the reach (figure 6D–F), similar to the fall in activation of both muscles observed in the shoulder. Unlike the shoulder, the dominant action of the elbow is extension. Although the early phase of the reach is similar to the shoulder, the elbow remains in quadrant I for the majority of the reach, indicating that its main role is to produce power to move the arm towards the target. In the final homing-in stages of the reach (figure 6D–F), the joint remains isometric as it stabilizes the arm about the target position.

**Figure 7.**
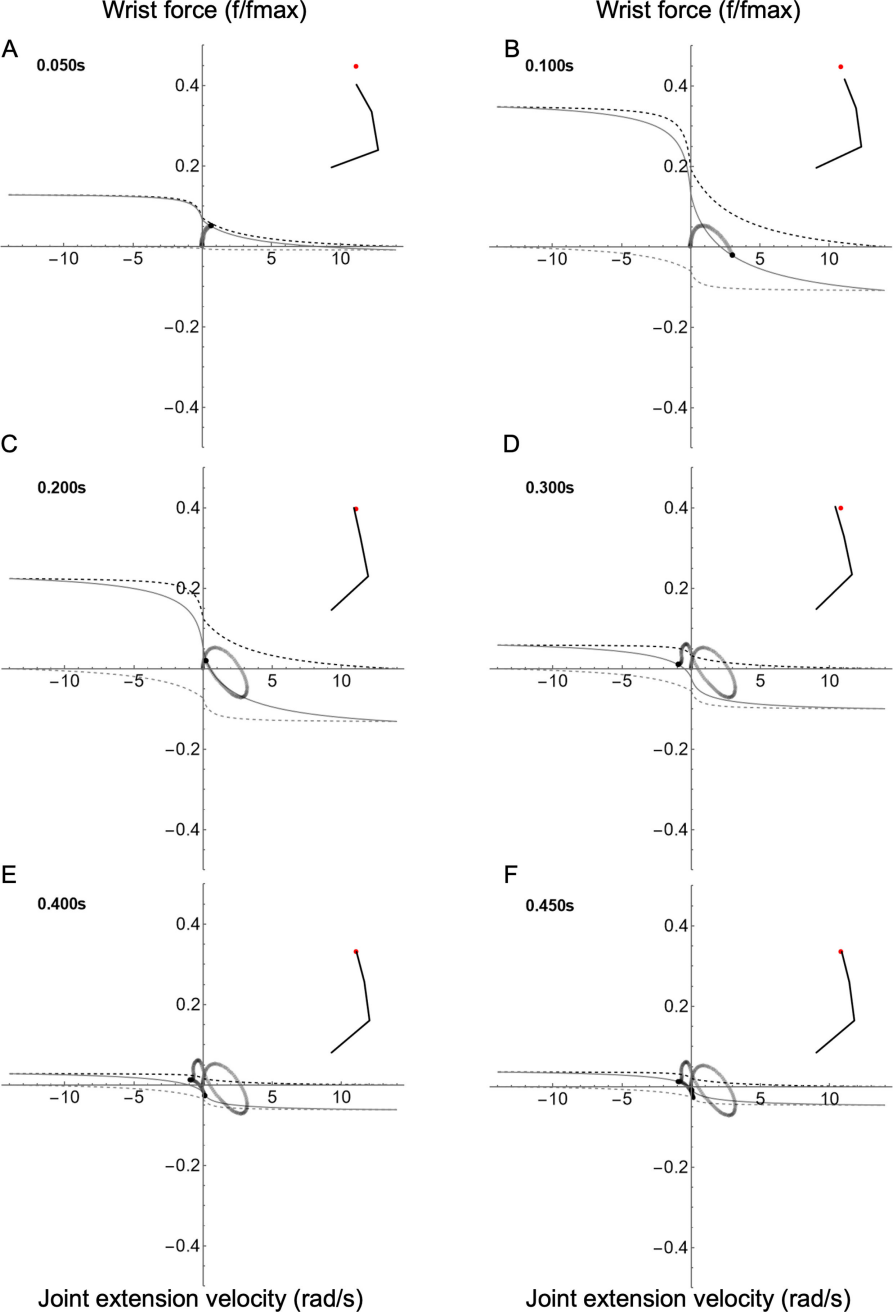
Joint–FV space animation for the wrist joint. FV curves are shown for six time frames throughout the reach (A–F). For the wrist, the extensor is defined as the agonist; therefore, positive force causes extension. The inset shows the position of the right arm relative to the target (red dot). The joint state trajectory is shown as a trail of grey dots, with the current state indicated by the black dot. The solid black line is the current joint–fv curve, and upper and lower limits of the joint–fv space are indicated by the dashed black and grey lines, respectively.

The wrist resembles the other joints in terms of the rise and fall of the size of the joint–fv space (figure 7; electronic supplementary material, Video S3). Like the elbow, the dominant movement of the wrist is extension. Additionally, the wrist functions like the elbow regarding its trajectory through the joint–FV space.

### 3.4. Comparing joint-level force–velocity dynamics of forward versus sideways versus cross-body reaching

**Figure 8.**
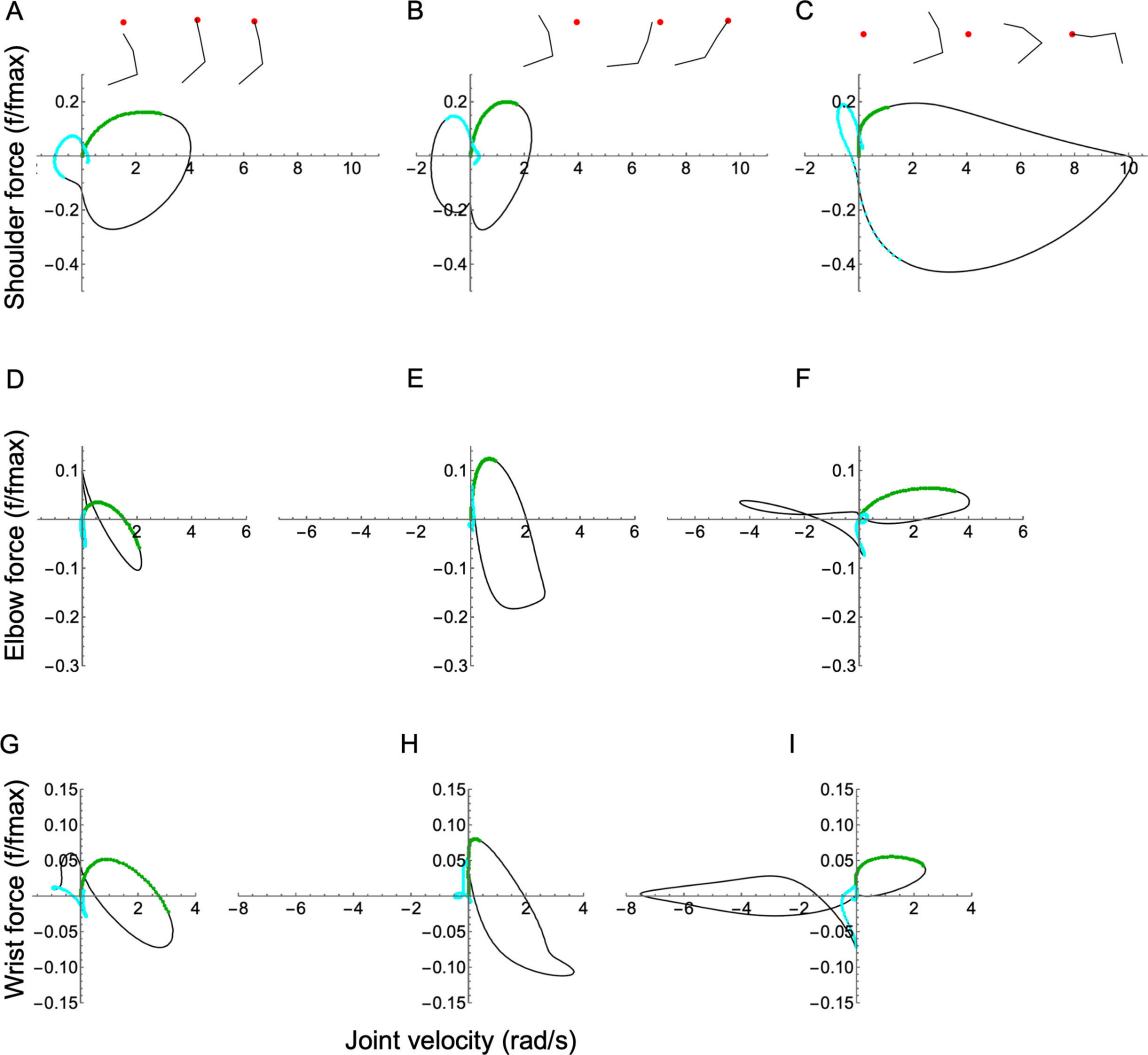
Joint–fv trajectories for the shoulder (top), elbow (middle) and wrist (bottom). The left column shows the forward reach (A,D,G), the middle column is the sideways reach (B,E,H) and the right column is the cross-body reach (C,F,I). Top insets show stick animation frames of the right arm and target (red dot). The thick green curve segment highlights the first 0.1 s of simulations, and the thick cyan highlights the final 0.2 s of simulations.

Comparison of forward, sideways and cross-body reaching showed broadly similar joint–fv trajectories; however, some unique features emerged depending on joint and reach type (figure 8; electronic supplementary material, Videos S3–S5). Overall, we identified three qualitative categories of joint–fv trajectory: (i) the circular-like path where the quadrants are traversed in sequence with a primary phase in quadrants I and IV and an overshoot-corrective phase in quadrants III and II (figure 8A–C), (ii) the ‘tied loop’ path where the loop starts to cycle through quadrants I and IV as above, but returns to the first quadrant before homing in, thus creating a ‘tie’ at the beginning/end of the loop (figure 8D,E,G,H) and (iii) the ‘fish-like’ path where the early reach is similar to other paths, but the latter half of the reach has an elongated ‘tail’ in quadrants II and III as the joint moves strongly in the opposite direction (figure 8F,I). Type 1 is exemplified by the shoulder joint, which is the most strikingly similar across reaches; its circular clockwise path indicates that joint movement is mostly driven by the shoulder muscles and characterized by agonist-powered acceleration in one direction, followed by antagonist braking and then a corrective movement in the opposite direction. This corrective movement is the smaller secondary loop (‘ear’) in quadrants II and III (figure 8A–C). In contrast, the elbow and wrist joints all showed more complex joint–fv trajectories, including anti-clockwise loops or isometric contractions, which are due to complex dynamics of inter-segment effects rather than contraction of the muscles moving the joint (see §4).

Despite the individual complexities in the above shapes, some insight can be gained by comparing them to the simple ‘idealized’ single-joint case (figure 4). Specifically, the circular-like trajectories in the first category are more similar to the single-joint case because the underlying dynamics of the shoulder more closely resemble a simple two-muscle-mass system. In contrast, the second and third shape categories reflect external forces (i.e. when the acceleration of the mass cannot be fully explained by joint torque).

### 3.5. The effects of a small mechanical perturbation on joint-level force–velocity dynamics

**Figure 9.**
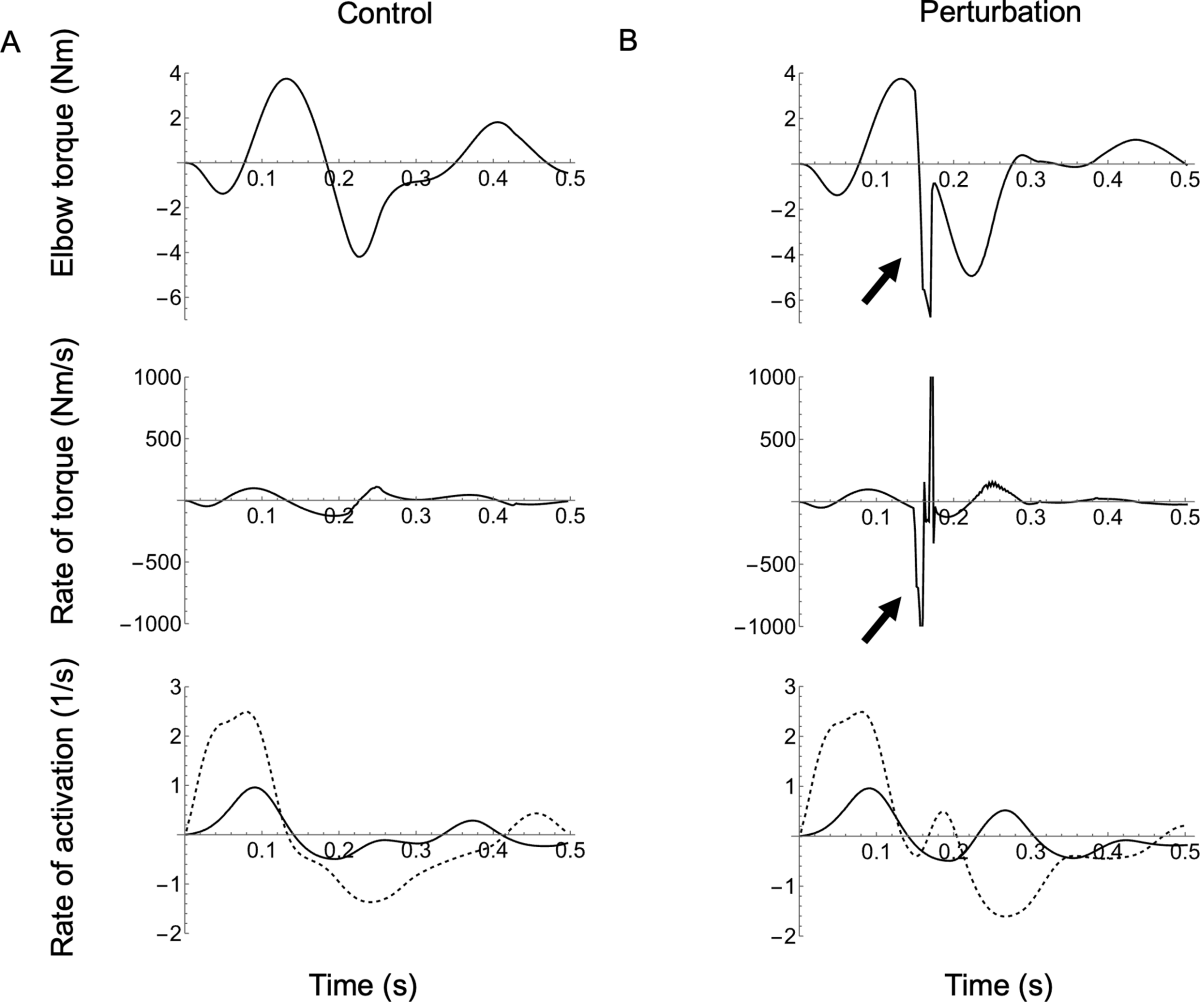
The effect of perturbation. Elbow joint torque, rate of torque (black) as well as the rate of activation (red) for the flexor (solid) and extensor (dashed) for control (A) versus perturbation (B) conditions. The arrows indicate the spike in torque and torque rate due to the perturbation. Note how, during the perturbation response, the transient rate of torque exceeds the single-muscle limit of activation rates and likely emerges from extremely rapid movement along the FV curve (see text).

**Figure 10.**
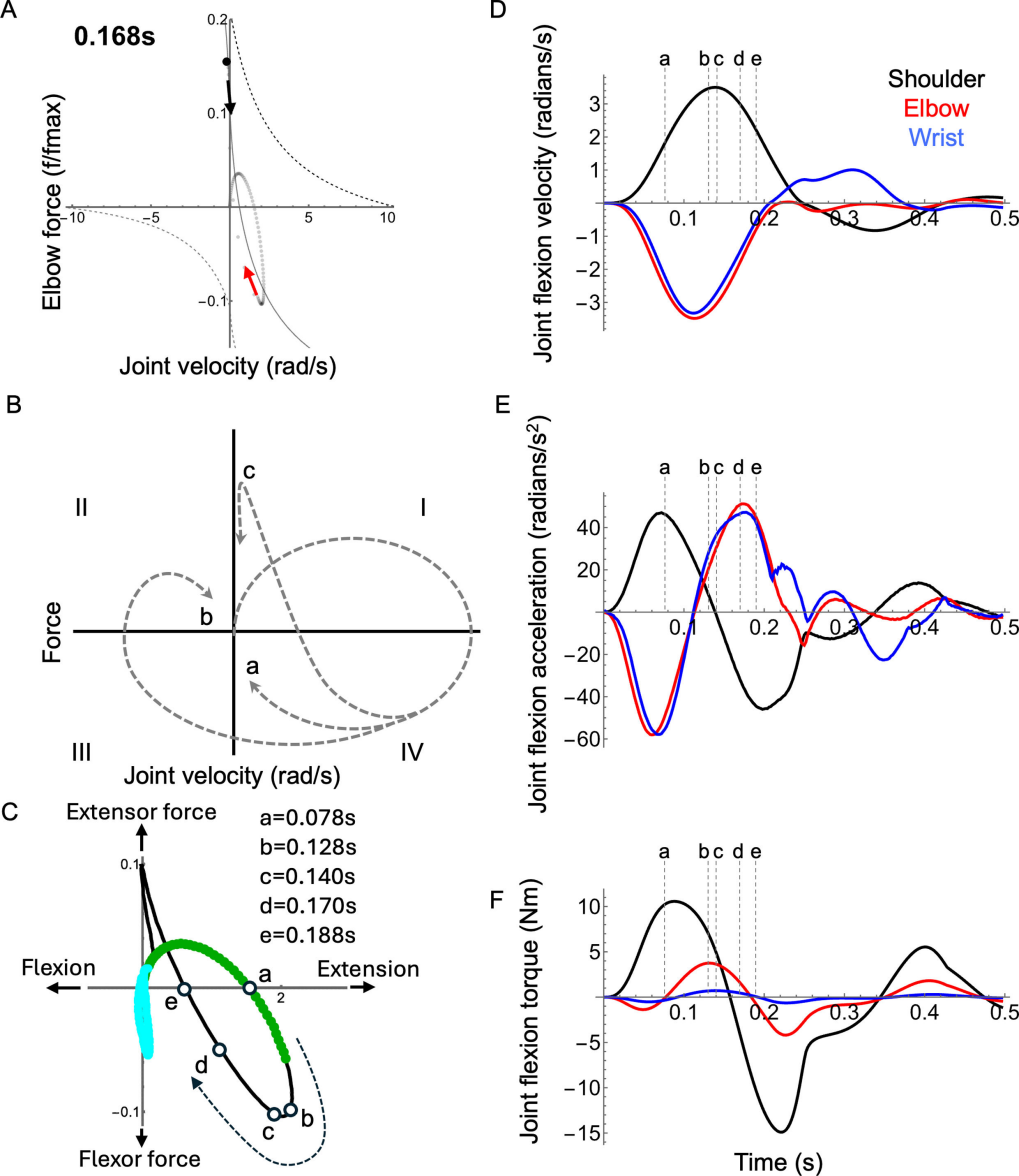
Effects of external forces. (A) A snapshot is shown from the elbow joint–fv trajectory at the instant just following the perturbation (same axes as in figure 5). At the perturbation, the joint is pushed upwards/leftwards along its joint–fv curve (red arrow). After the perturbation, the joint rebounds rapidly back down the region of the joint–fv curve where the slope (mechanical damping) is highest (black arrow). The black dot is the current joint state. (B) Idealized joint–fv trajectories for non-perturbed reaching (as in figure 2) showing (A) a perfectly planned single-joint path, (B) a feedback-corrected path similar to shoulder trajectories, and (C) a path with an ‘external force’ due to multi-joint interaction torques similar to elbow and wrist trajectories (see text). (C) The elbow trajectory (axes and colours as in figure 5A) with white circles showing trajectory time points of interest (a–e mark time points of 0.078, 0.128, 0.14, 0.17, 0.188 s) to aid the description in the text (see Discussion). The thick green curve segment highlights the first 0.1s of simulations and the thick light cyan highlights the final 0.2s. (D) Angular velocity, (E) acceleration and (F) net joint torque for shoulder (black), elbow (red) and wrist (blue) joints during the forward reach (unperturbed). Dashed vertical lines correspond to time points in panel (C). Note how the shoulder torque roughly follows joint acceleration. In contrast, elbow and wrist acceleration are dominated by cross-joint interaction torques and, consequently, the joint accelerations deviate from the net muscle torques, causing the joint trajectory to follow path c rather than a or b (panel B).

Reaching kinematics were slightly altered in the presence of a small mechanical perturbation; however, the impact was not strong enough to affect the reaching outcome (electronic supplementary material, Video S2). The effects of perturbation varied depending on the joint; however, we show the effects at the elbow because they are most pronounced and best illustrate the utility of the joint–FV visualization. The perturbation caused a minor dip in the elbow torque followed by a correction to allow the arm to reach the target successfully (figure 9A versus 9B; electronic supplementary material, Video S6). There were two phases of the correction stage. First, a rapid rebound phase returned the torque to its planned trajectory. This response can be seen in the extremely rapid movement of the joint’s position in joint–FV space. Second, a slower adjustment (mediated by the controller) modulated muscle activation to produce torque to compensate for the increased error during the perturbation. Differences between control and perturbation are most obvious when viewing the torque rate (i.e. its time derivative). Most strikingly, a rapid rebound correction occurs despite only modest changes in activation rate and force-length gain (additionally, moment arms are nearly constant). This suggests that FV effects play a large role in the most rapid part of the perturbation response. Specifically, the co-contracting muscles produced a joint–fv curve with a steeper slope than either muscle acting alone (figure 10A), possibly enabling the joint to respond more rapidly than a ‘controlled’ response.

## 4. Discussion

We aimed to develop a visual representation of joint mechanics in terms of joint-FV properties. To demonstrate this novel representation, we ran simulations with a previously published reaching model [31] and plotted the data in the joint–FV coordinate system. For ease of discussion, we adopt the useful abstraction that joints themselves ‘have actions’; i.e. a joint can be described as producing torque, work or power (see [36]) with the implicit understanding that a ‘joint’ in the musculoskeletal modelling sense is not an active tissue structure, but represents the net behaviour of all muscle and tendon actions crossing it.

There are three main results, corresponding to the questions posed in the introduction: (i) For any of the three representative reaches, the shoulder, elbow and wrist functioned similarly to each other in terms of gross joint kinetics. During the first phase, agonist muscles accelerated the arm towards the target. In the second phase, the antagonist muscles engaged to slow the limb, to minimize overshoot and to home in on the target (figures 5–7). Beyond these gross similarities, the shoulder functioned differently from either elbow or wrist. Specifically, the shoulder acted more like an isolated joint whose motion corresponds tightly to the net torque produced by its muscles. This was not the case for the elbow or wrist, whose movement patterns were strongly influenced by shoulder movement in addition to local muscle torque (see below). (ii) Joints functioned differently for the three reach targets. However, the qualitative differences were greater among joints than between reaching targets, especially for the shoulder compared to the elbow and wrist (figure 8). (iii) Co-contraction caused a steepening of the instantaneous joint–fv curve (figure 10A) enabling a damping-mediated rebound from small perturbations (figure 9B).

### 4.1. The joint-level force–velocity trajectory is a simple and concise portrait of joint mechanical function

The joint–fv trajectory is extremely rich with information. This information can be read at two levels. At the most basic level, the joint–fv trajectory can be read as a visual description of joint behaviour (regardless of the joint–fv curve shape); it is a portrait of joint kinetic function throughout a movement. For example, during the sideways reach, the shoulder action can be divided into four phases with extension during the first two phases (right quadrants) and then flexion during the final two (left quadrants). Over this motion pattern, the shoulder undergoes an alternating cycle of generating mechanical power (positive torque for acceleration; quadrant I) to absorbing mechanical power (negative torque for deceleration; quadrant IV) to generating and then absorbing power again (quadrants III and II, respectively; figure 8B). The first and second half of the reach correspond roughly to the submovements of reaching (see [37]), where the arm initially gets close to the target (primary submovement) then performs corrections and homing-in movements (secondary submovements).

Additionally, we view the joint–fv trajectory analogously to a work loop plot [12,13]. In this view, the joint–fv trajectory portrays the net muscle function about a joint and indicates how dominant muscles’ mechanical energy contributes towards net joint function (i.e. behaving like what is traditionally called a motor versus a strut versus a brake; see [16]). For example, the shoulder acts like a flexion motor during the early phase of reaches (until approx. 100 ms) driven by the dominant motor behaviour of the flexor. During the middle phase of reaches, the shoulder acts as a brake to slow the joint (action dominated by braking by the extensor), then later reverses to an extension motor to correct the overshoot of shoulder flexion. Likewise, the elbow and wrist also begin as extensor motors, but the elbow differs dramatically from shoulder function during the homing-in phase. Rather than reversing direction, the elbow produces an early braking torque, then functions isometrically (like a strut; see figure 6C–F where the joint–fv trajectory moves vertically along the *y*-axis). The wrist shares behaviour of both elbow and shoulder, showing early braking function (elbow-like) followed by late-stage extension-flexion reversal (shoulder-like).

At a second level, we go beyond a basic kinetic or energetic description by considering the underlying muscle force–velocity dynamics to explain *why* the joint behaves as observed. Although the traditional motor-brake metaphors [16] are useful at the single-muscle level, joint-level function requires more nuanced language to capture the joint’s mechanical state. For example, all joints might be traditionally called ‘motors’ during the first approximately 100 ms of reaches (clockwise trajectory in quadrant I), but the shoulder generates movement mainly through pure agonist action while the elbow and wrist modulate mechanical power production through co-contraction, as visualized by the size and location of the joint–fv space. This co-contraction makes the wrist and elbow ‘stiffer’, or more precisely, the relative actions of agonist and antagonist modulate the mechanical impedance (stiffness and damping) of the joint, as further discussed below.

A single muscle is considered to act as a brake when it operates on the lengthening region of the FV curve, but in a functional context, such as in the current study, braking rarely occurs in isolation. Instead, it often follows motoring, in the current study resulting in a relatively high co-activation in the muscle pair during what might be traditionally called ‘braking’ at the joint level. This is seen most prominently in the shoulder (figure 5C), but it is also visible in the elbow and wrist (figures 6B and 7B), although braking action of the elbow and wrist is complex (see below). As in the motor case, co-contraction can increase the impedance of the joint during braking action. This increased impedance is visible by observing the size of the joint–fv space, which allows a visual reading of the instantaneous ‘stiffness’ state of a joint. The examples above illustrate how the single-muscle view of ‘motor’ or ‘brake’ may be misleading at the joint level; one cannot strictly determine how much a muscle acts as a motor against the external load (e.g. limb inertia) versus against the antagonist. This ambiguity arises particularly in multibody systems where cross-joint interactions break the direct link between net muscle torque and joint movement (as is the case with the elbow; see below).

### 4.2. The shape and direction of the joint-level force–velocity trajectory help reveal the nature of underlying mechanical forces

To understand how joint–FV information links to underlying joint mechanics, we first note that the joint–fv trajectory is a description of muscle-produced torques, yet movement is governed by net joint torques. Consequently, the joint–fv trajectory may reveal situations where muscles produce a net torque, but the joint movement does not correspond to the direction of this net torque. While counterintuitive, this reveals information about the underlying dynamics of the whole system, as explored further below.

For an isolated joint moving a constant purely inertial load, one expects a direct correspondence between acceleration and torque. However, in multibody systems, limb segments move not just when muscle forces are acting directly on them, but also when upstream (i.e. more proximal) segments are moving [28]. The angular velocities and accelerations of upstream joints give rise to local fictitious forces (centrifugal, Coriolis and Euler forces), which cause movement in downstream joints because joint angles are defined relative to the axis of the previous (rotating) segment. Additional multibody effects also arise when the changing joint positions cause the inertial properties of the limb to change.

To illustrate these principles, we focus on the forward reach as a representative example. For a single-joint reach with no correction or homing-in, the joint follows a simple path through joint–FV space where increasing velocity (rightward motion in joint–FV space) corresponds to positive torque, whereas leftward movement corresponds to negative torque (figure 4H; path a figures 2D and 10B). During forward reaching, the joint–fv trajectory of the shoulder is a similar path, although it includes a ‘correction loop’ (path b; figures 2D and 10B). This is expected, as the shoulder has no upstream joints and the change in the arm position is small enough not to cause major inertial changes. Net muscle torques at the elbow and wrist, however, do not correspond to their respective accelerations (figure 10E,F), instead diverging dramatically from paths a and b. Notably, at time ≈ 0.14 s the elbow joint-fv trajectory veers sharply upwards/leftwards, indicating increasing (positive) torque while decelerating (figure 10C; electronic supplementary material, Video S3). The wrist shows a similar effect, though less pronounced (figure 7C; electronic supplementary material, Video S3). This elbow joint–fv pattern is counterintuitive for two reasons: (i) the joint is decelerating even when net muscle torque is positive and increasing (rather than accelerating as would be expected in a single-joint system), and (ii) activation of both muscles is decreasing while total elbow torque increases. Both of these observations suggest the presence of forces arising from outside the elbow muscles, specifically cross-joint interaction forces from the shoulder. These interaction forces can cause the total net torque on a joint to act in the opposite direction to the net muscle torque.

For the elbow, this cross-joint interaction occurs in three stages (figure 10C; electronic supplementary material, Video S3): (i) At the onset, the elbow extensor torque drives positive (extension) elbow acceleration (rightward movement in quadrant I, shown by the thick green segment of the trajectory curve). This extension occurs despite the opposing flexor torque caused by antagonism. (ii) At time ≈ 0.078 s (figure 10C point a) the flexor actions of the antagonist exceed the agonist action; the net torque action becomes flexion. However, despite the net flexor torque, the elbow continues to extend with increasing velocity until approximately 0.128 s (figure 10C point b), and this continued extension is due to multibody forces that counteract and exceed the net muscle action. Next, the joint begins to decelerate while the net muscle flexion torque increases, until approximately 0.140 s when the net flexion torque starts to decrease and the trajectory reverses direction back towards quadrant I (figure 10C points c and d). (iii) From time ≈0.188 s (figure 10C point e), the elbow crosses the *x*-axis again, returning to quadrant I, continuing to extend with increasing net extension torque, but counterintuitively, with diminishing speed. This behaviour indicates that while the net muscle torque would cause extension (extensor force exceeds flexor force), the net multibody torques work in the direction of flexion. During this stage, these multibody torques (mostly from shoulder deceleration) exceed elbow muscle torques, causing overall elbow deceleration. If not for these cross-joint effects, the elbow would follow a path similar to the shoulder (i.e. path b; figures 2D and figure 10B).

In light of the complex effects of cross-joint interactions described above, how useful are simple functional labels, such as motor and brake? We propose that these labels can be particularly useful in cases of clear separation between ‘sources and sinks’ of mechanical energy (such as a muscle in a swimming fish transferring momentum to the water; e.g. [38,39]) or a clear temporal separation between propulsion and braking (such as jumping versus landing in birds; e.g. [40,41]. However, in other cases, the flow of mechanical energy between muscle and load may be less clear. In our reaching example above, the elbow extensor might be called a ‘motor’ from the standpoint of mechanical energy; i.e. it shortens while producing force for the duration of its activity. This single-muscle perspective might suggest that its mechanical energy production contributes to the acceleration of a mass, but the muscle is mainly resisting combined forces of the antagonist and interaction torques from other joints. Hence, the ‘motor/brake’ metaphors may not always be precise enough to capture the true mechanical state of muscles in the anatomical context of multiple joints and antagonistic muscle pairs.

### 4.3. Co-contraction influences the shape of the joint-level force–velocity curve, which may influence the response to perturbations, but further exploration is needed

Even when cross-joint effects do not dominate movement, the mechanical state of a joint can be influenced strongly by antagonist co-contraction. Given that co-contraction naturally occurs during reaching (e.g. [42]) and may be an emergent response to excitation–contraction delays [31], we aimed to investigate how co-contraction influences joint dynamics. Prior simulation work found that the intrinsic damping of a single muscle’s FV relationship helps to stabilize cyclic hopping motions [43]. This intrinsic damping is most evident on the lengthening portion of the FV curve where an active muscle can absorb mechanical energy like a damper (e.g. from the recoil of a tendon following a landing impact; [40]). In theory, this damping ability becomes stronger during co-contraction because the antagonistic joint–fv curves add to steepen the local gradient, especially near-zero velocity (figure 1B). This steepening indicates that any change in joint velocity is strongly counteracted by changes in the net muscle torque, which is most visible at the elbow for both normal reaching (figure 6B,C) and the perturbation (figure 10A). During the perturbation simulation, this enhanced damping is responsible for the rapid rebound, which returns the joint torque nearly to pre-perturbation levels (figure 9B). We note that although the current work is not a rigorous perturbation study (see [44]), this simulated rapid rebound is a transient mechanical response which may augment other intrinsic stabilizing features of muscle, such as short-range stiffness [45], and thus warrants further investigation beyond the current scope. Additionally, given that co-contraction has been observed as an adaptive response to novel force fields [46], future work could address how co-contraction impacts task adaptation via changes in joint impedance arising from joint–FV dynamics.

### 4.4. Despite the current limited scope, the joint-level force–velocity framework is not limited to simple ‘idealized’ joints

For simplicity, our model (based on [31]) only contains one agonist–antagonist pair per joint and neglects both tendons and biarticular linkages. This level of complexity is sufficient for the current work because our main goal was to highlight conceptual insights gained by observing how FV properties interact with multibody dynamics. However, the conceptual joint–FV framework is not limited to simple models. As our framework utilizes the mathematical dependency between muscle contraction speed and joint angular velocity to transform muscle-specific FV curves to the joint coordinate system, it can be generalized to account for additional curves from additional muscle actions. Furthermore, our framework allows muscles with unique properties (*v_max_*, shape of the FV curve, moment arms, etc.) to be summed (equivalently to equation (2.4)) to create a multi-muscle, multi-articular joint–fv curve. Finally, the effect of tendons could be incorporated in future studies, as long as tendon properties are known. We note, however, that for the current work, tendons are unlikely to impact our results because the muscle forces (therefore tendon displacements) are relatively small. Furthermore, the current work only aims to introduce the framework and to highlight the rich information contained in joint–FV data. Hence, we feel that the addition of further complexity (multiple muscles with varying properties) is beyond the current scope and will be addressed in subsequent studies.

Further to the above limitations, we did not investigate the effects of varying force–velocity properties. Importantly, the shape of the FV curve is known to vary significantly across species (e.g. in frogs; [47]) and among fibre types within a species (e.g. in mice; [48]), and it has been found to strongly impact muscle function in musculoskeletal models [49]. We note, however, that given the current focus on how generalized FV properties impact joint function, concerns about specific variation in FV parameters would not add meaningfully to the presented findings. However, a study of the influence of FV shape on joint–FV dynamics would be a fruitful area of future investigation, especially if joint–FV dynamics were to be analysed comparatively across species.

Finally, we acknowledge that Hill-type models lack stiffness and history-dependent effects (see [50,51]) and therefore have limited utility for studying transient behaviour such as general responses to perturbations. Hence, we restricted our perturbation to small forces (see §2), where the behaviour of Hill-type models remains reasonable. Moreover, we focused most of our analysis on movements where transient behaviour is not dominant. Regardless, more advanced models (e.g. [52]) would enable a thorough future study of how muscle impedance influences transient responses to perturbations. Although the joint–FV framework would need to be reworked for such models, the key concept still applies: multiple muscle-level states can be condensed into instantaneous joint-level representations by transforming them into the joint coordinate system.

## 5. Summary and conclusion

We developed a conceptual framework for visualizing and describing how muscle force–velocity properties influence joint dynamics. In our investigation of simulated human reaching, we used joint-FV plots to compare the dynamic function of the shoulder, elbow and wrist across three different reach trajectories. We found broad similarities across all joints and conditions, especially during the early phase of reaching. However, the elbow and wrist differed from the shoulder during the homing-in phase, which showed a complex interplay of local muscle-driven torque and interaction torques due to shoulder movement. Additionally, we found that co-contraction creates a steepening of the joint–fv curve (compared with a single-muscle fv curve), which may confer increased damping and self-correction from small mechanical perturbations. More broadly, our new visualization of joint–fv trajectories communicates joint kinetic function in the context of muscle activation and force–velocity dynamics that underlie this behaviour. Although joint–FV plots (like work loops) discard time information, they can be used as tools to guide decisions about which time-series data are most valuable to observe in detail. Furthermore, time information could be incorporated in future studies either as a third dimension or encoded with colour for a compact representation of joint-level muscle dynamics. We therefore propose that our joint-FV framework can be used to explain the intricate features seen in muscle data from musculoskeletal simulations, enabling a better understanding of how intrinsic muscle properties limit the performance of dynamical systems.

## Data Availability

All source code to run the simulations as well as simulation data, code for data analysis and code for figure generation are available in GitHub: https://github.com/frogtronics/jointFV_RSOS and have been archived within the Zenodo repository: [53]. Supplementary material is available online [54].
